# Systemic immune-inflammation index is associated with hepatic steatosis: Evidence from NHANES 2015-2018

**DOI:** 10.3389/fimmu.2022.1058779

**Published:** 2022-11-18

**Authors:** Yancheng Song, Wencong Guo, Zhaopeng Li, Dong Guo, Zhao Li, Yu Li

**Affiliations:** ^1^ Department of Gastrointestinal Surgery, The Affiliated Hospital of Qingdao University, Qingdao, Shandong, China; ^2^ Laboratory of Nephrology & Department of Nephrology, The Affiliated Qingdao Municipal Hospital of Qingdao University, Qingdao, Shandong, China

**Keywords:** systemic immune-inflammation index (SII), hepatic steatosis, non-alcoholic fatty liver disease, bariatric surgery, NHANES

## Abstract

**Background:**

As a novel inflammatory marker, Systemic Immune-Inflammation Index (SII) has not been studied with hepatic steatosis. The aim of this study was to investigate the possible relationship between SII and hepatic steatosis.

**Methods:**

In the cross-sectional investigation, adults having complete information on SII, hepatic steatosis, and bariatric surgery from the 2015–2018 National Health and Nutrition Examination Survey (NHANES) were included. Hepatic steatosis was evaluated with heaptic steatosis index (HSI). The platelet count × neutrophil count/lymphocyte count was used to compute SII. We investigated the independent interaction between SII and hepatic steatosis using weighted multivariable regression analysis and subgroup analysis. To explore the potential relationship between SII, bariatric surgery and hepatic steatosis by controlling potential confounders by propensity score matching.

**Results:**

The study involved 10505 participants in total, 5937 (56.5%) of whom had hepatic steatosis according to the diagnosis. After adjusted for covariates, multivariable logistic regression revealed that high SII level was an independent risk factor for hepatic steatosis (OR = 1.30, 95% CI: 1.10-1.52, P 0.01). Unexpectedly, bariatric surgery reduced SII even after PSM corrected for differences of BMI and HSI.

**Conclusions:**

In US adults, SII was positively correlated with an increase in hepatic steatosis. The SII may be a simple and affordable way to identify hepatic steatosis. Bariatric surgery may reduce SII without resorting to weight loss. This needs to be verified in additional prospective research.

## Introduction

Hepatic steatosis, an accumulation of fat in the liver that is usually linked to obesity, can proceed to fibrosis, cirrhosis, and nonalcoholic fatty liver disease (NAFLD) (1). The etiology and accompanying conditions, such as inflammation and fibrosis, which can cause cirrhosis and liver failure, affect the natural course of hepatic steatosis (2). Given the rising incidence of obesity globally, the deleterious effects hepatic steatosis are becoming a growing challenge for public health. The deleterious effects of hepatic steatosis are becoming a growing challenge for public health due to the rising incidence of obesity globally (3). NAFLD is now the most common type of hepatic steatosis, affecting 30%-40% of male and 15%-20% of female in the general population. It is regarded as a hepatic manifestation of metabolic syndrome and is connected to insulin resistance, hypertension, atherosclerosis, obesity, dyslipidemia. Nonalcoholic steatohepatitis (NASH), which can progress to cirrhosis, is caused by lipid buildup in hepatocytes, which causes oxidative stress and an inflammatory response. As a result, hepatic steatosis should be given special clinical attention.

Hu et al. created the systemic immune-inflammation index (SII) in 2014 to reflect local immune response and systemic inflammation across the human body (4). Studies have discovered that in malignant tumors patients, SII can objectively represent the balance between the inflammatory reaction and immunological response (5, 6). SII is currently utilized as a prognostic indicator in the research of carcinoma (7–9). However, research on SII in disorders like steatosis that affect the chronic liver is scarce.

Numerous investigations have demonstrated that inflammation is a crucial factor in hepatic steatosis (3, 10). Hepatic steatosis may proceed to hepatocyte damage, the development of inflammation, and the activation of immune cells, or it may remain benign. Infiltrating macrophages, T lymphocytes, neutrophils, and DCs are examples of the inflammatory cells in the liver that cause inflammation (11). However, it is not yet clear how the inflammatory level biomarker SII and hepatic steatosis are related.

Therefore, to ascertain the relationship between SII levels and hepatic steatosis among participants in the US National Health and Nutrition Examination Survey (NHANES), we carried out a population-based investigation.

## Materials and methods

### Data and sample sources

Data were downloaded from the National Health and Nutrition Examination Survey (NHANES), a nationally representative cross-sectional survey designed and conducted by the National Center for Health Statistics (NCHS). The survey samples the U.S. population using a stratified, multistage probability approach and offers health and nutrition statistics on the non-institutionalized civilian population in the United States. The NCHS Research Ethics Review Board authorized the survey, verifying that all participants provided informed permission. Detailed statistics can be accessible at https://www.cdc.gov/nchs/nhanes/.

**Figure 1:**
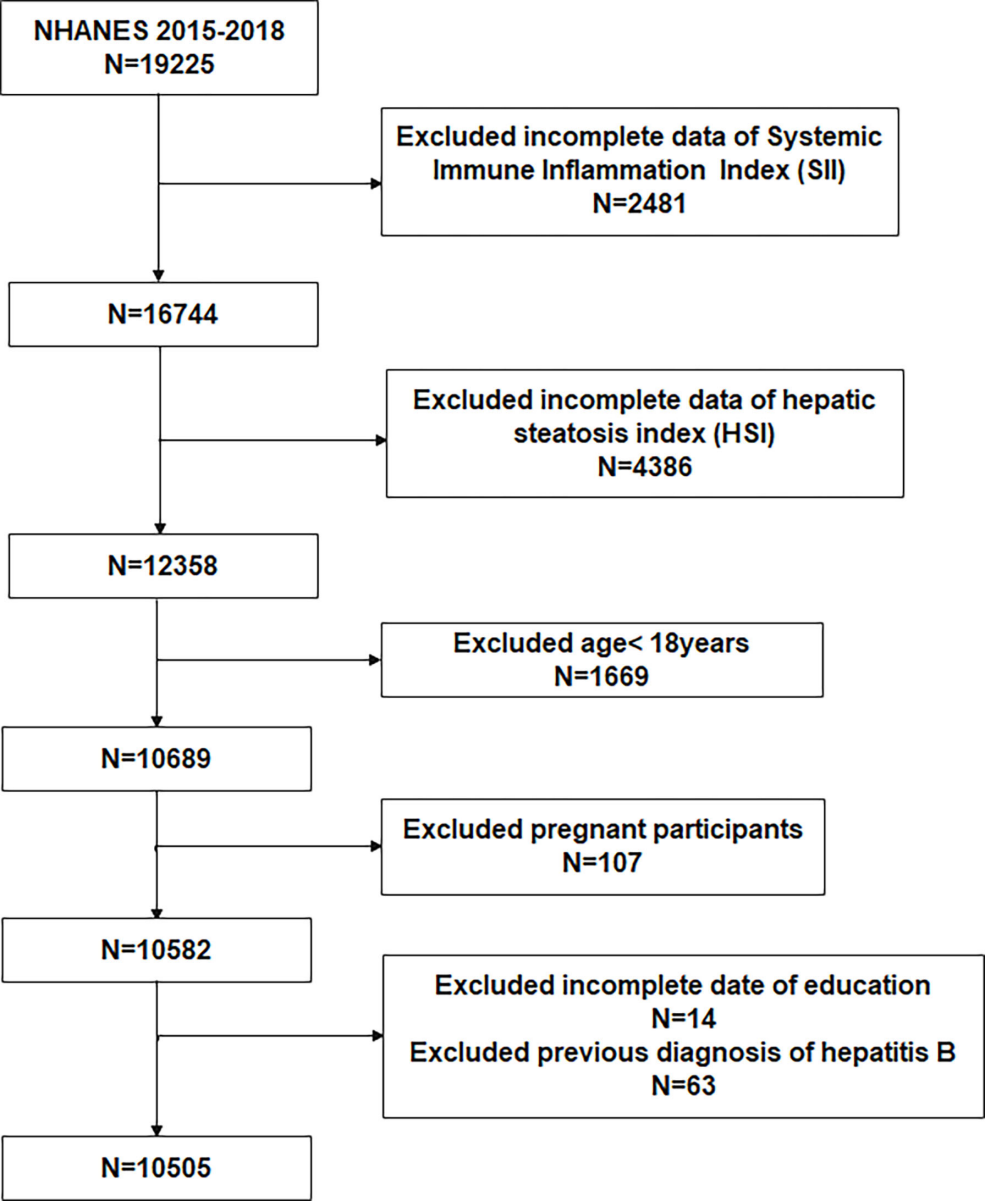
Flowchart of the participants selection from NHANES 2015–2018.

To evaluate the participants’ nutritional and physical health, standardized in-home interviews, physical examinations, and laboratory tests were carried out at mobile examination centers. The study included adults (age ≥ 18) in the NHANES 2015-2018 cycle. 19,225 participants were involved. Of these participants, we excluded 2481 with missing SII, 4386 without HSI,1669 with age < 18, 107 with pregnant, 14 with missing education, and 63 with hepatitis B. Eventually, 10,505 participants were enrolled in the study (**Figure 1**).

### Exposure variable

Lymphocyte, neutrophil, and platelet counts (expressed as ×10^3^ cells/μl) were measured using automated hematology analyzing devices. The following formula is utilized to calculate SII: (platelet count × neutrophils count)/lymphocytes count (4, 12).

### Outcome variable

The degree of hepatic steatosis was assessed by HSI. The SII levels were calculated using the following formula: 8 ×(alanine aminotransferase (ALT)/aspartate aminotransferase (AST) ratio) + body mass index (BMI) +2 (if diabetic) + 2 (if female) (13). Previous research has demonstrated a significant link between HSI and the degree of hepatic steatosis, and NAFLD is frequently regarded as having an HSI > 36 (13, 14). Thus, HSI=36 was used as a cutoff value to assess hepatic steatosis.

### Covariates

This investigation included covariates that may impact the relationship between SII and hepatic steatosis. Demographic parameters included age, sex, race, education level, smoking, BMI, systolic blood pressure (SBP), and diastolic blood pressure (DBP), and ratio of family income to poverty (PIR). Total cholesterol (TC), triglycerides (TG), and glycohemoglobin were included in the biochemical profile. Health risk factors included diabetes, hyperlipidemia, and hypertension.

### Statistical analyses

Given the complex sampling survey, weighted analyses were performed according to the recommendations of the NHANES. The weighted student’s t-test (continuous variables) or weighted chi-square test (categoric variables) were utilized to compare differences in baseline characteristics between the normal and hepatic steatosis groups. According to the Youden index, the optimal cutoff value of SII level was determined by using receiver operating characteristics curve (ROC). Multivariate logistic regression analysis was used to evaluate the correlation between SII and hepatic steatosis in different models. Model 1: confounding variables were not adjusted. Model 2: age, race, education, and smoking status were adjusted. Model 3: age, sex, race, PIR, education levels, smoking status, diabetes, hyperlipidemia, hypertension, ALT, AST, glycohemoglobin, TC, TG, SBP, DBP were adjusted. To investigate the relationship between SII and hepatic steatosis in different subgroups, subgroup analysis was carried out. Stratification factors included gender (male/female), age (<60/≥60 years), hypertension (yes/no), diabetes (yes/no), hyperlipidemia (yes/no). Interaction analysis was used to evaluate the heterogeneity of the association between the subgroups. This study further investigated the influence of bariatric surgery on the relationship between SII and hepatic steatosis. Propensity score matching (PSM) was utilized to eliminate bias and control for potential confounding variables. The “MatchIt” package of R was used for PSM analysis. The “nhanesR” package was used to extract and analyze data. P<0.05 was considered statistically significant.

## Results

### Baseline characteristics of participants

A total of 10505 participants were involved, with an average age of 49.28 years and a gender split of 48.62% men to 51.38% women; 56.52% participants were categorized as having hepatic steatosis. The 10505 participants represented 229.7 million non-institutionalized civilian population of the United States. Hepatic steatosis in patients was different with statistical significance of age, race, education, BMI, diabetes mellitus (DM), smoking status, hypertension, hyperlipidemia, SII, ALT, glycohemoglobin, TC, TG, SBP, and DBP (all p<0.05). Sex, poverty income ratio, and AST did not differ between patients with and without hepatic steatosis. The clinical and biochemical characteristics of the participants are shown in **Table 1**. The optimal SII cut-off value was 445.210 (AUC: 0.542). The ROC curve was shown in **Supplementary Figure S1**.

**Table 1:** Basic characteristics of participants (n = 10505) in the NHANES 2015–2018.

Outcomes	Normal (n=4568)	hepatic steatosis (n=5937)	P-value
Age	46.20 ± 0.60	48.77 ± 0.46	< 0.001
Sex			0.97
Female	51.44 (49.23,53.64)	51.37 (49.69,53.05)	
Male	48.56 (46.36,50.77)	48.63 (46.95,50.31)	
PIR	3.02 ± 0.06	2.96 ± 0.06	0.3
Race			< 0.0001
Mexican American	6.13 (4.26, 7.99)	11.64 (8.29,14.99)	
Non-Hispanic White	65.30 (60.70,69.90)	61.86 (57.12,66.60)	
Non-Hispanic Black	10.58 (8.14,13.01)	11.22 (8.40,14.03)	
Other Hispanic	5.87 (4.42,7.31)	7.34 (5.75,8.93)	
	12.13 (9.77,14.49)	7.94 (6.54, 9.34)	
Education			0.002
> High school	22.90 (20.41,25.40)	26.31 (24.51,28.12)	
< High school	12.59 (10.79,14.39)	13.48 (11.49,15.47)	
Above	64.51 (60.98,68.03)	60.20 (57.36,63.05)	
BMI (kg/m^2^)	23.79 ± 0.07	33.88 ± 0.15	< 0.0001
Diabetes Mellitus			< 0.0001
Yes	6.53 (5.82, 7.25)	23.10 (21.37,24.84)	
No	93.47 (92.75,94.18)	76.90 (75.16,78.63)	
Smoke			0.003
Former	22.19 (20.27,24.11)	26.47 (24.76,28.19)	
Never	58.72 (56.13,61.32)	57.13 (55.23,59.03)	
Now	19.09 (16.74,21.43)	16.40 (14.91,17.89)	
Hypertension			<0.0001
Yes	27.73 (25.55,29.91)	47.33 (44.82,49.84)	
No	72.27 (70.09,74.45)	52.67 (50.16,55.18)	
Hyperlipidemia			<0.0001
Yes	54.21 (51.07,57.35)	76.55 (74.60,78.51)	
No	45.79 (42.65,48.93)	23.45 (21.49,25.40)	
SII	506.11 ± 9.27	530.29 ± 6.07	0.02
ALT (U/L)	19.20 ± 0.28	28.06 ± 0.31	< 0.0001
AST (U/L)	23.63 ± 0.29	24.23 ± 0.27	0.17
Glycohemoglobin (%)	5.44 ± 0.02	5.86 ± 0.02	< 0.0001
TC (mmol/L)	4.82 ± 0.03	4.97 ± 0.04	< 0.001
TG (mmol/L)	1.32 ± 0.02	1.93 ± 0.04	< 0.0001
SBP (mmHg)	120.51 ± 0.48	125.65 ± 0.33	< 0.0001
DBP (mmHg)	69.61 ± 0.42	72.82 ± 0.36	< 0.0001

Mean ± SD was for continuous variables. The percentage (95% confidence interval) was for categorical variables.

NHANES, National Health and Nutrition Examination Survey; PIR, poverty income ratio; BMI, body mass index; SII, systemic immune-inflammation index; SBP, systolic blood pressure; DBP, diastolic blood pressure; ALT, alanine aminotransferase; AST, aspartate aminotransferase; TC, total cholesterol; TG, triglycerides.

### SII is an independent risk factor for hepatic steatosis

After adjusting for other potential confounding factors, we created a number of models to evaluate the independent effects of SII on hepatic steatosis. In univariate analysis, age, race, education levels, DM, smoking status, hypertension, hyperlipidemia, SII, ALT, glycohemoglobin, TC, TG, SBP, and DBP were connected with a higher incidence of hepatic steatosis (p < 0.05, **Supplementary Table S1**). According to logistic regression analysis, revealed that SII levels were independently related to hepatic steatosis (OR = 1.000, 95% CI: 1.000–1.001, p = 0.041). In univariate analysis, high levels of SII were a risk factor for hepatic steatosis (OR = 1.42, 95% CI: 1.26–1.59, P < 0.0001, **Table 2**). After adjustment for age, sex, race, poverty income ratio, education levels, smoking status, DM, hyperlipidemia, hypertension, ALT, AST, glycohemoglobin, SBP, DBP, TC, TG, high SII levels were an independent risk factor for hepatic steatosis (OR = 1.30, 95% CI: 1.10–1.52, P < 0.01).

**Table 2:** Association Between SII and hepatic steatosis.

SII	OR	95% CI	P
Model 1^a^			
< 445.21	Reference		
≥ 445.21	1.42	1.42 (1.26,1.59)	< 0.0001
Model 2^b^			
< 445.21	Reference		
≥445.21	1.43	1.43 (1.27,1.61)	< 0.0001
Model 3^c^			
< 445.21	Reference		
≥445.21	1.30	1.30 (1.10,1.52)	< 0.01

^a^Model 1 did not adjust for any confounding factors. ^b^Model 2 adjusted for age, race, education, smoking status. ^c^Model 3 adjusted for age, sex, race, poverty income ratio, education, smoking status, DM, Hyperlipidemia, Hypertension, ALT, AST, glycohemoglobin, SBP, DBP, TC, TG.

SII, systemic immune-inﬂammation index; OR, odds ratio; CI, confdence interval.

### Subgroup analysis

**Figure 2:**
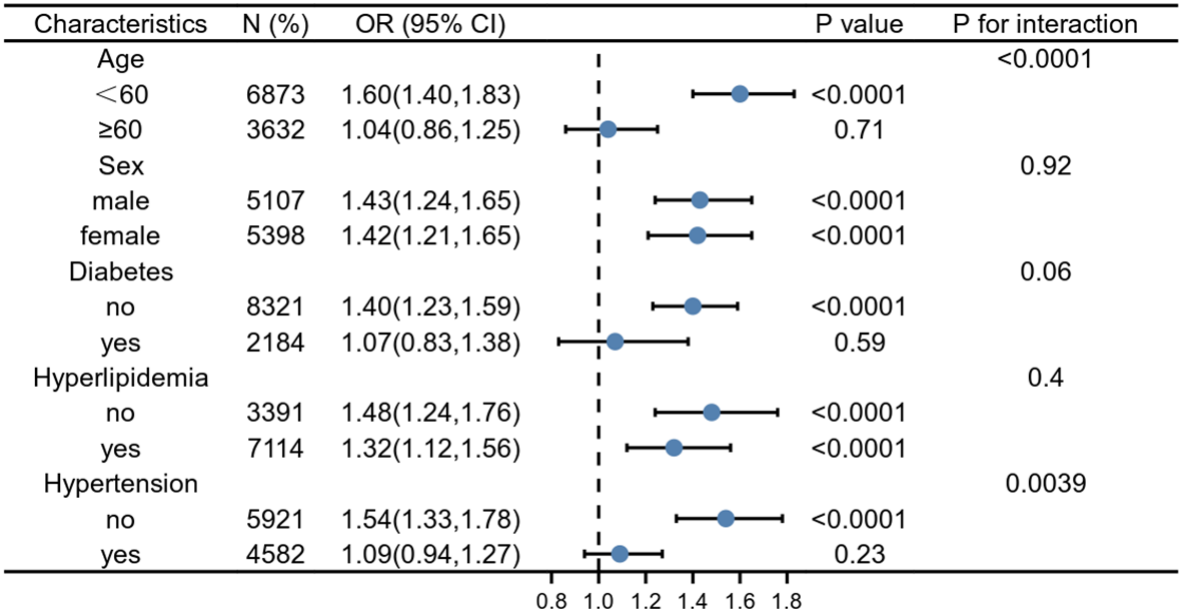
Subgroup analysis for the association between SII and hepatic steatosis. Weighted univariate logistic regression was used for subgroup analysis.

Our subgroup analysis results revealed that there were inconsistent relationships between SII level and hepatic steatosis (**Figure 2**). A significant association of SII with hepatic steatosis was observed in each subgroup for the subgroup stratified by sex and hyperlipidemia (all p<0.05). As for the subgroup stratified by age, diabetes, and hypertension, connection with statistical significance was only observed among those participants with age < 60 years, without diabetes and hypertension. Although not statistically significant (P >0.05), a positive association between SII and hepatic steatosis was observed in participants aged ≥60, with diabetes and hypertension. The interaction test revealed no significant differences among gender, diabetes, and hyperlipidemia in the relationship between SII and hepatic steatosis, demonstrating that these factors had no significant influence on this positive relationship (p for interaction >0.05). Contrary, age and hypertension may influence the positive association between SII and hepatic steatosis (p for interaction <0.05).

### PSM analysis

PSM analysis was conducted to evaluate the association between SII and bariatric surgery in participants with hepatic steatosis. Older, female, high education, high household income, and high BMI were more likely to receive bariatric surgery (**Supplementary Table S2**). These characteristics were not significantly different after PSM (**Table 3**). The baseline characteristics of patients in patients with/without bariatric surgery groups were shown in **Table 3**. In hepatic steatosis patients with/without bariatric surgery, there was different with statistical significance of hyperlipidemia, ALT, and TG. The rest characteristics did not differ between hepatic steatosis patients with and without bariatric surgery. Noteworthy, bariatric surgery reduced high SII levels associated with hepatic steatosis, independent of BMI.

**Table 3:** Basic characteristics of participants with hepatic steatosis with/without bariatric surgery after PSM Analysis in the NHANES 2015–2018.

Outcomes	hepatic steatosis without bariatric surgery (n=1080)	hepatic steatosis with bariatric surgery (n=108)	P-value
Age	52.88 ± 0.71	52.73 ± 1.26	0.92
Sex			0.46
Female	82.85 (79.93, 85.78)	84.14 (76.04, 92.25)	
Male	17.15 (14.22, 20.07)	15.86 (7.75,23.96)	
PIR	3.53 ± 0.07	3.54 ± 0.24	0.98
Race			0.83
Mexican American	6.57 (4.34,8.80)	5.95 (2.24,9.67)	
Non-Hispanic White	69.55 (64.34,74.75)	70.12 (62.73,77.50)	
Non-Hispanic Black	12.32 (8.60,16.05)	13.19 (8.20,18.19)	
Other Hispanic	4.20 (2.90,5.49)	5.00 (0.56,9.44)	
Other Race	7.37 (5.07, 9.66)	5.73 (0.68,10.78)	
Education			0.84
> High school	24.74 (21.48,28.00)	26.73 (14.80,38.66)	
< High school	2.60 (1.70,3.49)	2.51 (-0.14,5.15)	
Above	72.66 (69.40,75.93)	70.76 (58.25,83.27)	
BMI (kg/m^2^)	36.92 ± 0.29	37.61 ± 0.68	0.37
HSI	47.31 ± 0.34	46.82 ± 0.73	0.56
Diabetes Mellitus			0.5
Yes	26.27 (22.67,29.87)	31.24 (22.05,40.44)	
No	73.73 (70.13,77.33)	68.76 (59.56,77.95)	
Smoke			0.87
Former	28.53 (24.53,32.53)	21.66 (10.90,32.42)	
Never	60.83 (56.64,65.01)	61.03 (47.17,74.89)	
Now	10.64 (8.26,13.03)	17.31 (6.28,28.35)	
Hypertension			0.57
Yes	54.82 (50.09,59.54)	56.13 (39.88,72.38)	
No	45.18 (40.46,49.91)	43.87 (27.62,60.12)	
Hyperlipidemia			0.003
Yes	54.82 (50.09,59.54)	56.13 (39.88,72.38)	
No	21.70 (18.25,25.15)	34.19 (23.48,44.90)	
SII	567.80 ± 10.58	510.12 ± 18.84	0.01
ALT (U/L)	24.99 ± 0.55	19.76 ± 1.58	0.003
AST (U/L)	23.57 ± 0.67	21.74 ± 1.33	0.17
Glycohemoglobin (%)	5.96 ± 0.04	5.88 ± 0.14	0.61
TC (mmol/L)	4.82 ± 0.03	4.97 ± 0.04	0.19
TG (mmol/L)	1.78 ± 0.04	1.46 ± 0.11	0.01
SBP (mmHg)	127.34 ± 0.76	124.25 ± 1.29	0.05
DBP (mmHg)	72.24 ± 0.59	70.66 ± 1.03	0.19

Mean ± SD was for continuous variables. The percentage (95% confidence interval) was for categorical variables.

NHANES, National Health and Nutrition Examination Survey; PIR, poverty income ratio; BMI, body mass index; SII, systemic immune-inflammation index; SBP, systolic blood pressure; DBP, diastolic blood pressure; ALT, alanine aminotransferase; AST, aspartate aminotransferase; TC, total cholesterol; TG, triglycerides.

## Discussion

By reviewing the literature, this is the first study that identify the direct relationship between SII and hepatic steatosis. In our cross-sectional study, which included 10505 participants, we discovered that participants with hepatic steatosis had significantly higher SII levels, besides participants with hepatic steatosis underwent bariatric surgery had significantly lower SII than did those without bariatric surgery. Our findings revealed that elevated SII levels were an independently risk factor for hepatic steatosis.

Previous studies using various epidemiological methods and target groups have demonstrated the correlation between SII and liver disorders. An Indonesian population-based cohort of 196 patients with hepatocellular carcinoma (HCC) demonstrated that SII was a better accurate predictor of 1-year survival in patients with advanced HCC who did not receive treatment than neutrophil-lymphocyte ratio (NLR) (15). Elevated pre-treatment SII was correlated to lower overall survival (HR:1.54, P < 0.001) and earlier time to recurrence (HR:1.77, P < 0.001), according to a meta-analysis of ten published retrospective studies including 2796 HCC patients (16). For disorders of the metabolism, this is especially true. Among individuals from the 2011–2016 NHANES, a cross-sectional study discovered a statistically significant correlation between SII and higher BMI (17). Other NHANES studies suggested that higher SII could increase the risk of peripheral arterial disease, isolated coronary artery ectasia, and Diabetic depression (18–20). Likewise, similar to all this, our subgroup analysis indicated that participants with hepatic steatosis had a higher risk of high SII than participants without hepatic steatosis, suggesting that people with hepatic steatosis should be given more attention, especially in individuals with NAFLD.

The precise mechanism underlying the relationship between inflammation and hepatic steatosis is yet uncertain. According to previous research, in rats, visceral fat accumulation and hepatic inflammation are the important factors that promote the progression of steatosis (21). Patients with hepatic steatosis may proceed to hepatic fibrosis because of increased production of the proinflammatory cytokine tumor necrosis factor-alpha in the Kupffer cells, which enhances oxidative stress. Furthermore, TNF-a may cause hepatic cellular damage by increasing glomerular mononuclear cell infiltration, hence hastening the course of liver failure (22). Susanne et al. proposed that the causes of liver inflammation may originate outside the liver (for instance, in adipose tissue or the digestive tract) together with inside the organ (for example, lipotoxicity, innate immune responses, cell death pathways, mitochondrial dysfunction, and endoplasmic reticulum stress), which may be one of the potential mechanisms (23). In similarity to the majority of previous investigations, our research discovered that a higher SII was individually correlated with a higher risk of hepatic steatosis, indicating that SII may have an independently significant detrimental effect on hepatic steatosis.

Additionally, our results indicate that SII could be used as a straightforward, non-invasive indicator to determine high-risk patients in hepatic steatosis. However, there are various classical inflammatory markers with widespread therapeutic uses in clinical practice, such as neutrophil-to-lymphocyte ratio (NLR) and platelet-to-lymphocyte ratio (PLR). Canan et al. hypothesized that the rise in SII and NLR supports the increase in inflammation brought on by the rise of fat in children with obesity. In addition, SII and NLR imply that there might be inflammatory markers that can be employed in follow-up study for obesity comorbidities (24). Jin et al. found that higher NLR levels were negatively correlated with progressed inflammatory activity and substantial fibrosis in Chinese patients with NAFLD (25).

Currently, bariatric surgery is considered effective in reducing hepatic steatosis (26, 27). According to a prospective 5-year follow-up of obesity patients with NASH by Lassailly et al, the positive effects of bariatric surgery on the resolution of NASH were long-lasting and resulted in a sustained decrease in fibrosis (28). In our cross-sectional study, the decrease of SII supports the decrease of inflammation due to bariatric surgery in hepatic steatosis participants. This indirectly indicates that there is a strong association between SII and hepatic steatosis. Bariatric surgery, which leads to weight loss, is generally believed to be the main reason for the ameliorate of hepatic steatosis and inflammation (29). Unexpectedly, bariatric surgery reduced SII even after PSM corrected for differences of BMI and HSI. It suggests that bariatric surgery may reduce inflammation through other pathways without resorting to weight loss and hepatic steatosis, such as gut microbiota and modification of ghrelin (30, 31). In addition, the effect of bariatric surgery on the expression of immune-related genes and the abundance of inflammatory cell infiltration may also be one of the potential mechanisms (3, 32). To the best of our knowledge, this is the first study to evaluate the relationship among SII, bariatric surgery, and hepatic steatosis. Our study showed that SII may be an effective marker for assessing the efficacy of bariatric surgery.

Many studies have demonstrated that SII has remarkable predictive ability. Simultaneously, SII was a widely available method with a non-intrusive methodology, simple accessibility, and low cost. The potential for therapeutic use is indeed positive. Our research has its own advantages. First, the sample size is sufficient, and the sample selection is representative. Second, to get more trustworthy results, we adjusted for confounding variables. However, the study’s shortcomings call for cautious interpretation of the findings. First, the cross-sectional study design precluded us from establishing a causal association. Therefore, future research with a larger number of participants is still necessary to define the causal relationship. Furthermore, despite the fact that we made adjustments for several relevant confounders, we were incapable of totally rule out the impact of additional potential confounding variables.

## Conclusion

In summary, our study found that SII and hepatic steatosis were associated, and that bariatric surgery can decrease both SII and inflammation. To validate our findings, further expanded prospective investigations at scale are required.

## Data availability statement

Publicly available datasets were analyzed in this study. This data can be found here: https://www.cdc.gov/nchs/nhanes.

## Ethics statement

The studies involving human participants were reviewed and approved by the Research Ethics Review Board of the NCHS. The patients/participants provided their written informed consent to participate in this study.

## Author contributions

YS & YL put forward the conception and design of the study. YS & WG collected and analyzed the data. ZPL, DG and ZL made the tables and figures. all the authors drafted and revised the paper. All authors contributed to the article and approved the submitted version.

## Acknowledgments

Thanks to Zhang Jing (shanghai Tongren Hospital) for his work on the NHANES database. His outstanding work, nhanesR package and webpage, makes it easier for us to explore NHANES database.

## Conflict of interest

The authors declare that the research was conducted in the absence of any commercial or financial relationships that could be construed as a potential conflict of interest.

## Publisher’s note

All claims expressed in this article are solely those of the authors and do not necessarily represent those of their affiliated organizations, or those of the publisher, the editors and the reviewers. Any product that may be evaluated in this article, or claim that may be made by its manufacturer, is not guaranteed or endorsed by the publisher.
