# Supplementary material for: Systemic immune-inflammation index is associated with hepatic steatosis: Evidence from NHANES 2015-2018
Source: Front Immunol. 2022 Nov 18;13:1058779. doi: 10.3389/fimmu.2022.1058779 (PMC9718528; doi:10.3389/fimmu.2022.1058779)
Supplement: Supplementary file 1 [file DataSheet_1.docx]

Supplementary Material

# Supplementary Tables

**Supplementary Table S1** **Univariate logistic regression associating variables with hepatic steatosis.**

| Outcomes | OR (95%CI) *P* |
| --- | --- |
| Age | 1.008(1.004,1.012) <0.001 |
| Sex |  |
| Male | 1.003(0.881,1.142) 0.966 |
| Female | Reference |
| Race |  |
| Mexican Ameri | Reference |
| Other Hispanic | 0.658(0.531,0.817) <0.001 |
| Non-Hispanic White | 0.498(0.433,0.574) <0.0001 |
| Non-Hispanic Black | 0.558(0.467,0.667) <0.0001 |
| Other | 0.344(0.282,0.420) <0.0001 |
| Education |  |
| Primary school | Reference |
| High school | 1.073(0.931,1.236) 0.318 |
| Above | 0.871(0.768,0.989) 0.034 |
| Diabetes Mellitus |  |
| Yes | 4.298(3.669,5.035) <0.0001 |
| No | Reference |
| Smoke |  |
| Never | Reference |
| Former | 1.226(1.078,1.395) 0.003 |
| Now | 0.883(0.742,1.051) 0.155 |
| Hypertension |  |
| Yes | 2.342(2.041,2.688) <0.0001 |
| No | Reference |
| Hyperlipidemia |  |
| Yes | 2.758(2.365,3.216) <0.0001 |
| No | Reference |
| SII | 1.000(1.000,1.001) 0.041 |
| ALT (U/L) | 1.060(1.046,1.074) <0.0001 |
| AST (U/L) | 1.004(0.998,1.009) 0.201 |
| Glycohemoglobin (%) | 2.145(1.901,2.421) <0.0001 |
| TC (mmol/L) | 1.153(1.078,1.232) <0.001 |
| TG (mmol/L) | 1.829(1.673,1.999) <0.0001 |
| SBP (mmHg) | 1.018(1.014,1.022) <0.0001 |
| DBP (mmHg) | 1.027(1.021,1.033) <0.0001 |

Abbreviations: OR, odds ratio; CI, confidence interval; PIR, poverty income ratio; BMI, Body Mass Index.

**Supplementary Table S2** **Basic characteristics of participants with hepatic steatosis with/without bariatric surgery before matched analysis in the NHANES 2015–2018**

| Outcomes | P-OB | Z-BS | P-value |
| --- | --- | --- | --- |
| Age | 48.68±0.46 | 52.73±1.26 | 0.003 |
| Sex |  |  | < 0.0001 |
| Female | 50.63(48.87,52.39) | 84.14(76.04,92.25) |  |
| Male | 49.37(47.61,51.13) | 15.86(7.75,23.96) |  |
| PIR | 2.94±0.06 | 3.54±0.24 | 0.02 |
| Race |  |  | 0.16 |
| Mexican American | 11.77(8.40,15.14) | 5.95(2.24, 9.67) |  |
| Non-Hispanic White | 61.67(56.90,66.45) | 70.12(62.73,77.50) |  |
| Non-Hispanic Black | 11.17(8.34,14.00) | 13.19(8.20,18.19) |  |
| Other Hispanic | 7.39(5.78,9.01) | 5.00(0.56,9.44) |  |
| Other Race | 7.99(6.58, 9.40) | 5.73(0.68,10.78) |  |
| Education |  |  | 0.03 |
| > High school | 26.31(24.42,28.19) | 26.73(14.80,38.66) |  |
| < High school | 13.73(11.73,15.72) | 2.51(-0.14,5.15) |  |
| Above | 59.97(57.08,62.85) | 70.76(58.25,83.27) |  |
| BMI (kg/m^2^) | 33.80±0.15 | 37.61±0.68 | < 0.0001 |
| Diabetes Mellitus |  |  | 0.05 |
| Yes | 22.92(21.22,24.62) | 31.24(22.05,40.44) |  |
| No | 77.08(75.38,78.78) | 68.76(59.56,77.95) |  |
| Smoke |  |  | 0.74 |
| Former | 26.58(24.83,28.32) | 21.66(10.90,32.42) |  |
| Never | 57.04(55.17,58.91) | 61.03(47.17,74.89) |  |
| Now | 16.38(14.85,17.91) | 17.31(6.28,28.35) |  |
| Hypertension |  |  | 0.29 |
| Yes | 47.13(44.65,49.62) | 56.13(39.88,72.38) |  |
| No | 52.87(50.38,55.35) | 43.87(27.62,60.12) |  |
| Hyperlipidemia |  |  | 0.04 |
| Yes | 47.13(44.65,49.62) | 56.13(39.88,72.38) |  |
| No | 23.20(21.20,25.21) | 34.19(23.48,44.90) |  |
| SII | 530.74± 6.23 | 510.12±18.84 | 0.32 |
| ALT (U/L) | 28.24±0.32 | 19.76±1.58 | < 0.0001 |
| AST (U/L) | 24.29±0.27 | 21.74±1.33 | 0.06 |
| Glycohemoglobin (%) | 5.86±0.02 | 5.88±0.14 | 0.88 |
| TC (mmol/L) | 4.98±0.04 | 4.97±0.04 | 0.23 |
| TG (mmol/L) | 1.94±0.04 | 1.46±0.11 | < 0.0001 |
| SBP (mmHg) | 125.68±0.33 | 124.25±1.29 | 0.29 |
| DBP (mmHg) | 72.87±0.37 | 70.66±1.03 | 0.04 |

Note: Mean ± SD was for continuous variables. The percentage (95% confidence interval) was for categorical variables.

Abbreviations: NHANES, National Health and Nutrition Examination Survey; PIR, poverty income ratio; BMI, body mass index; SII, systemic immune-inflammation index; sbp, [systolic](https://cn.bing.com/dict/search?q=Systolic&FORM=BDVSP6&cc=cn) [blood](https://cn.bing.com/dict/search?q=blood&FORM=BDVSP6&cc=cn) [pressure](https://cn.bing.com/dict/search?q=pressure&FORM=BDVSP6&cc=cn); dbp, d[iastolic](https://cn.bing.com/dict/search?q=Diastolic&FORM=BDVSP6&cc=cn) [blood](https://cn.bing.com/dict/search?q=blood&FORM=BDVSP6&cc=cn) [pressure](https://cn.bing.com/dict/search?q=pressure&FORM=BDVSP6&cc=cn).

# Supplementary Figure

**Supplementary Figure S1 ROC curves of SII**


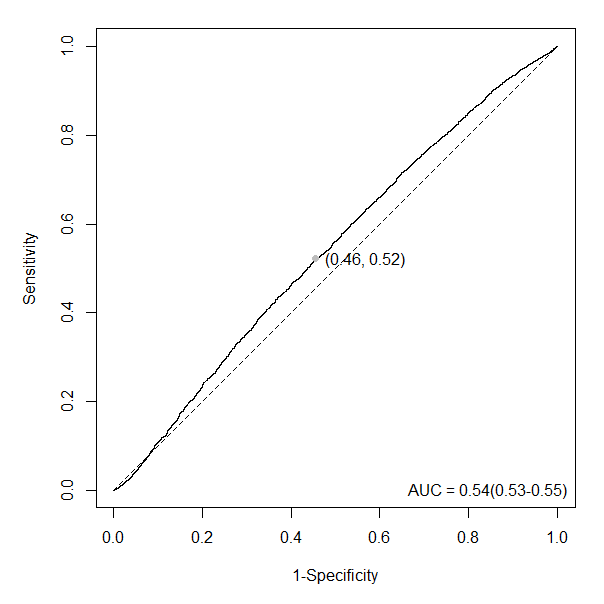


**
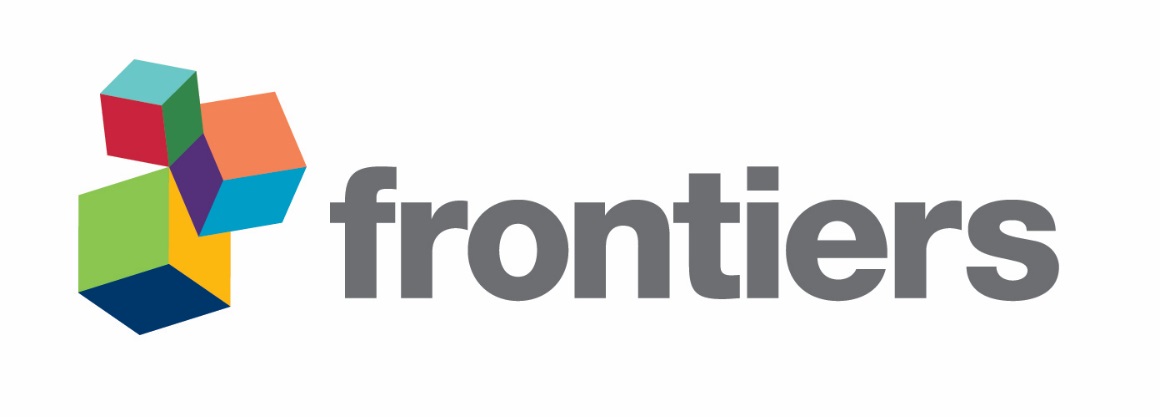
**
